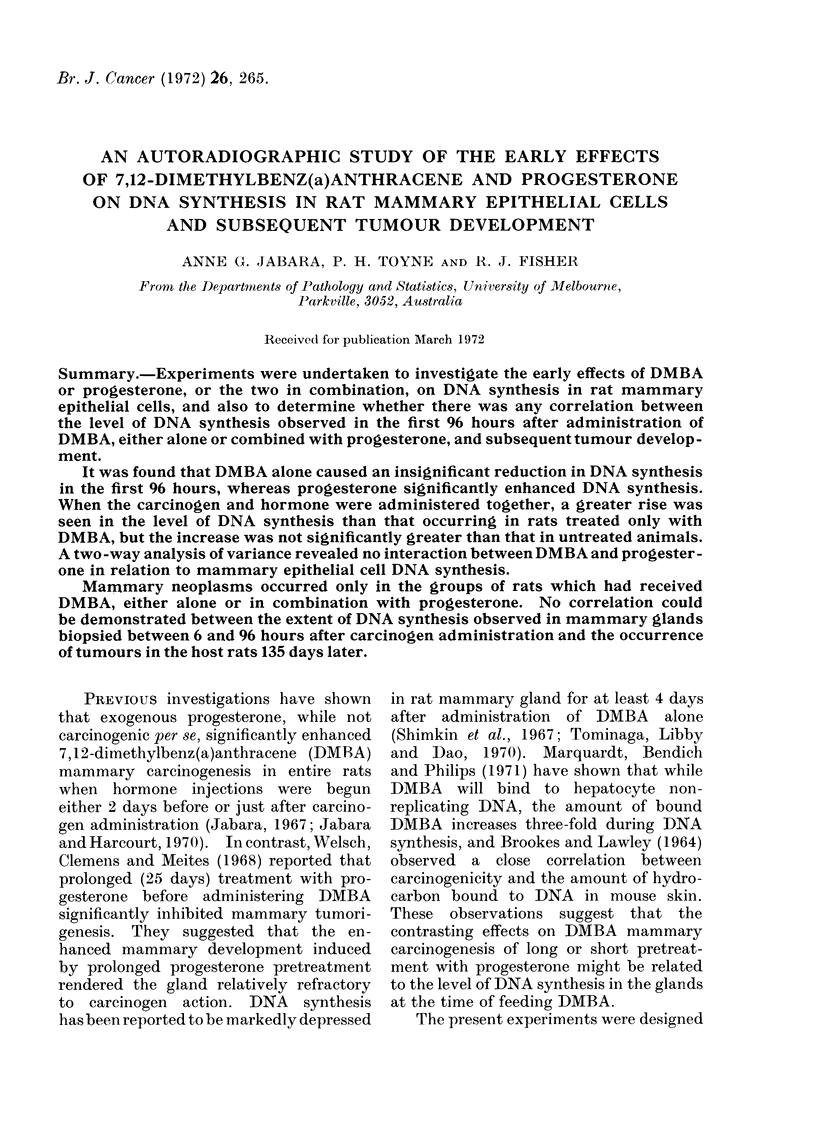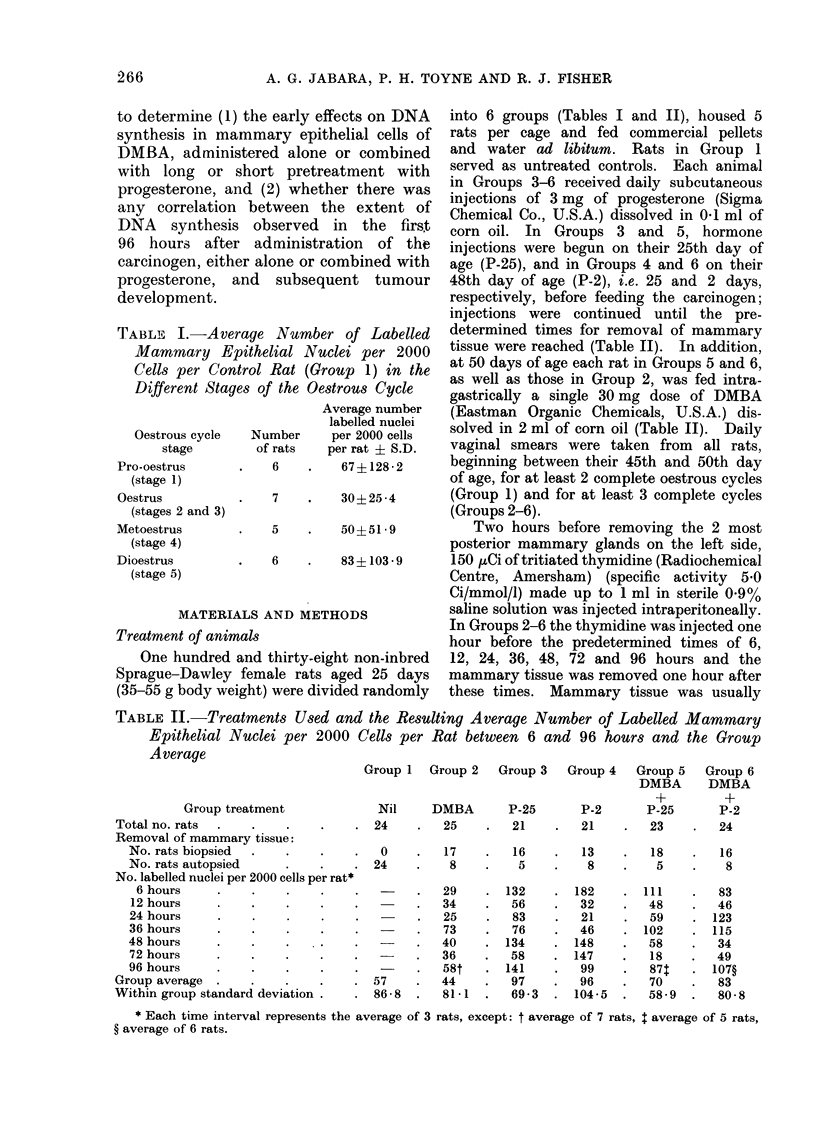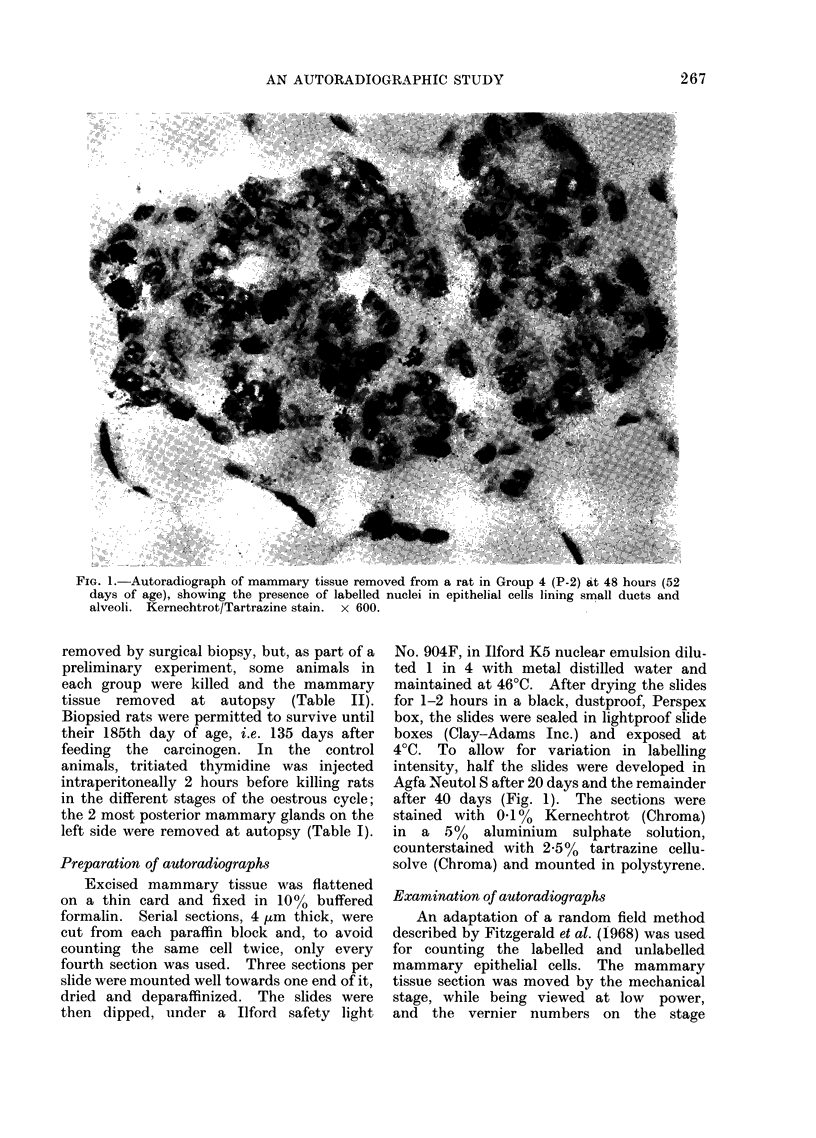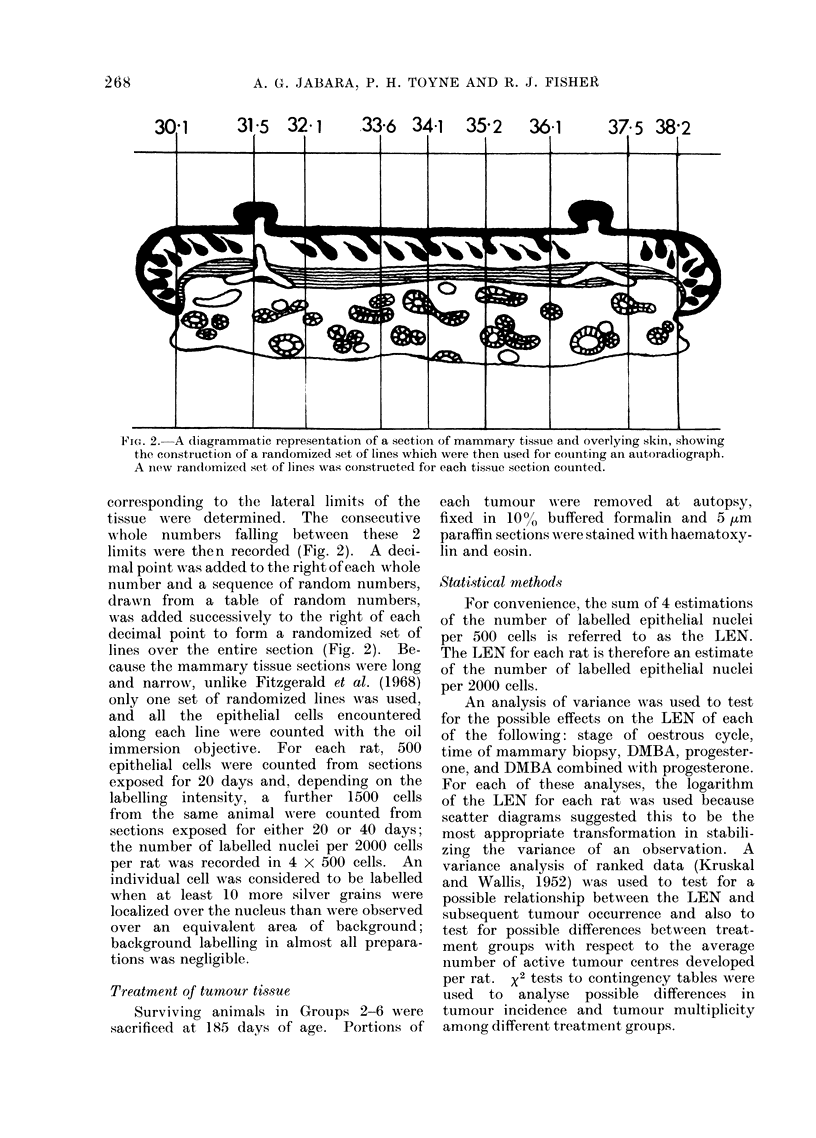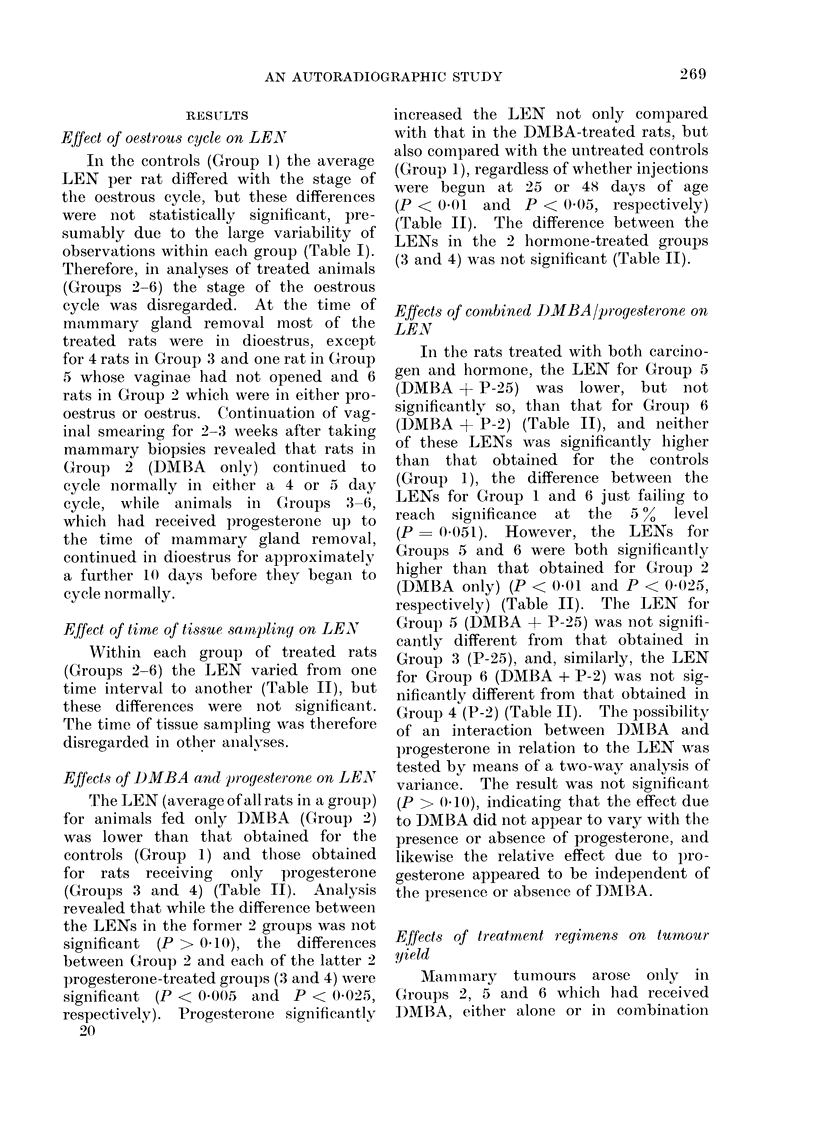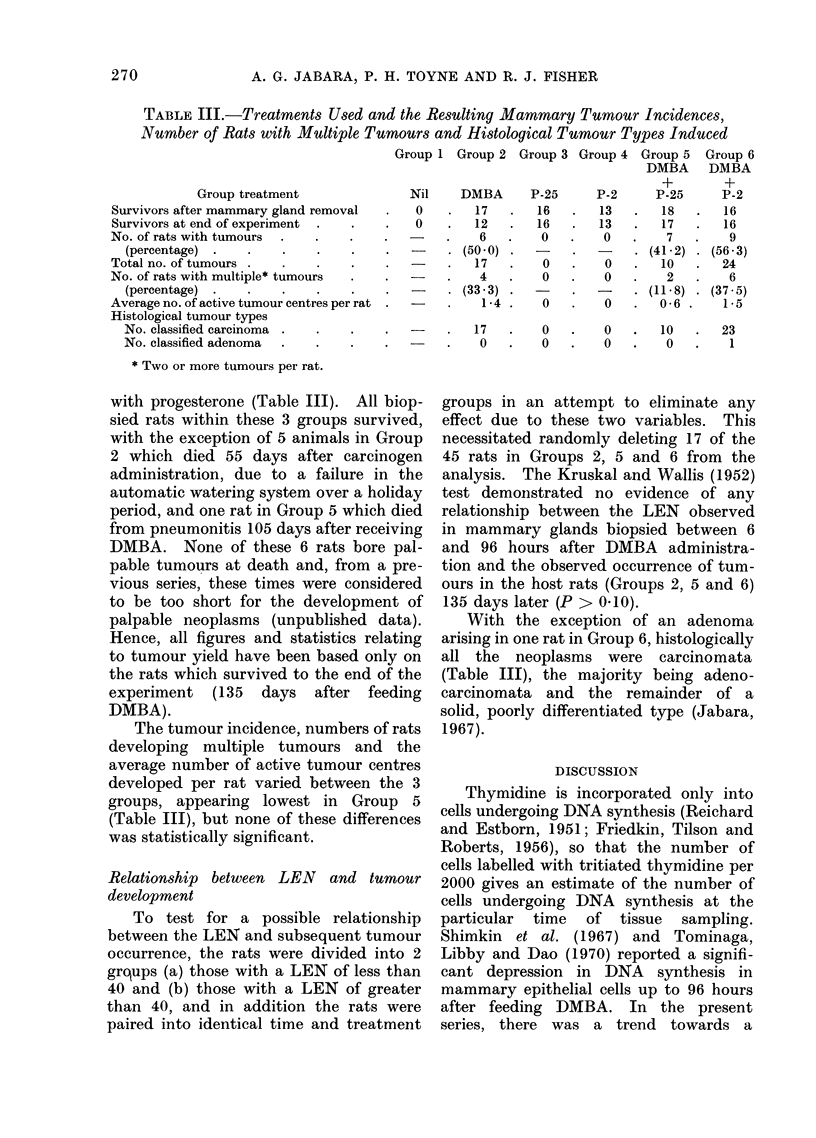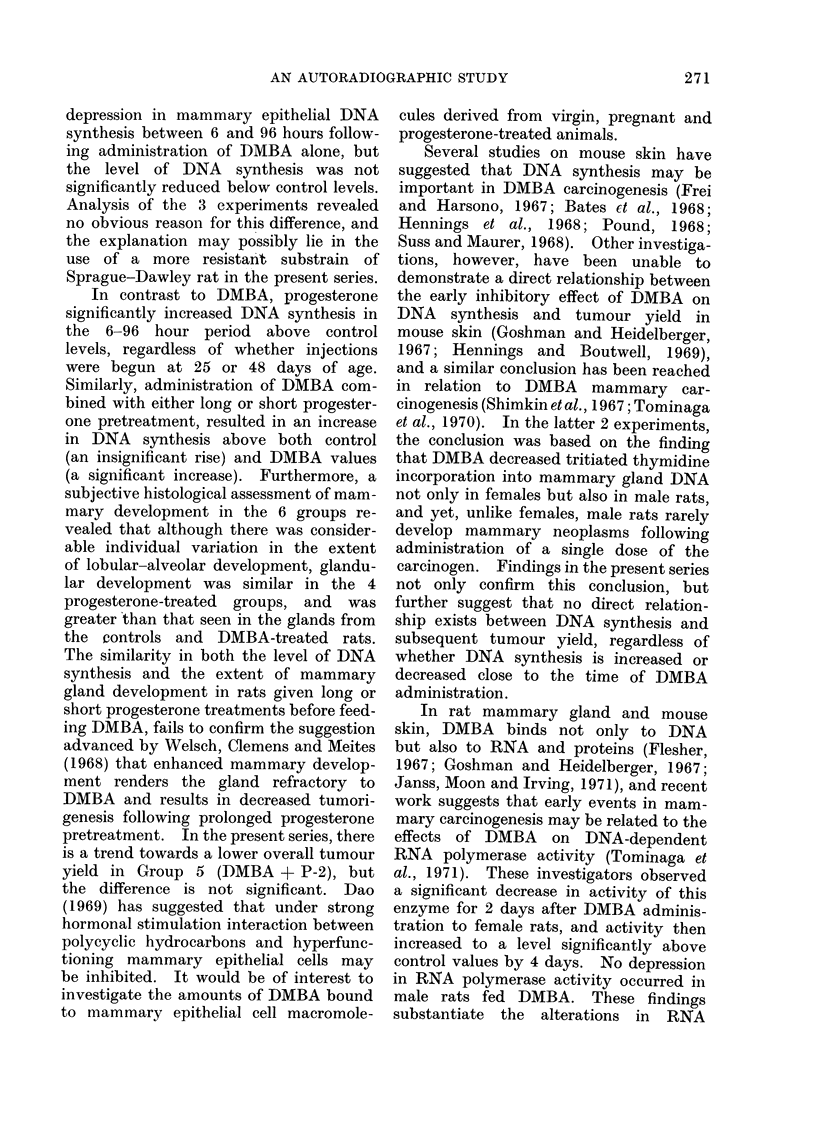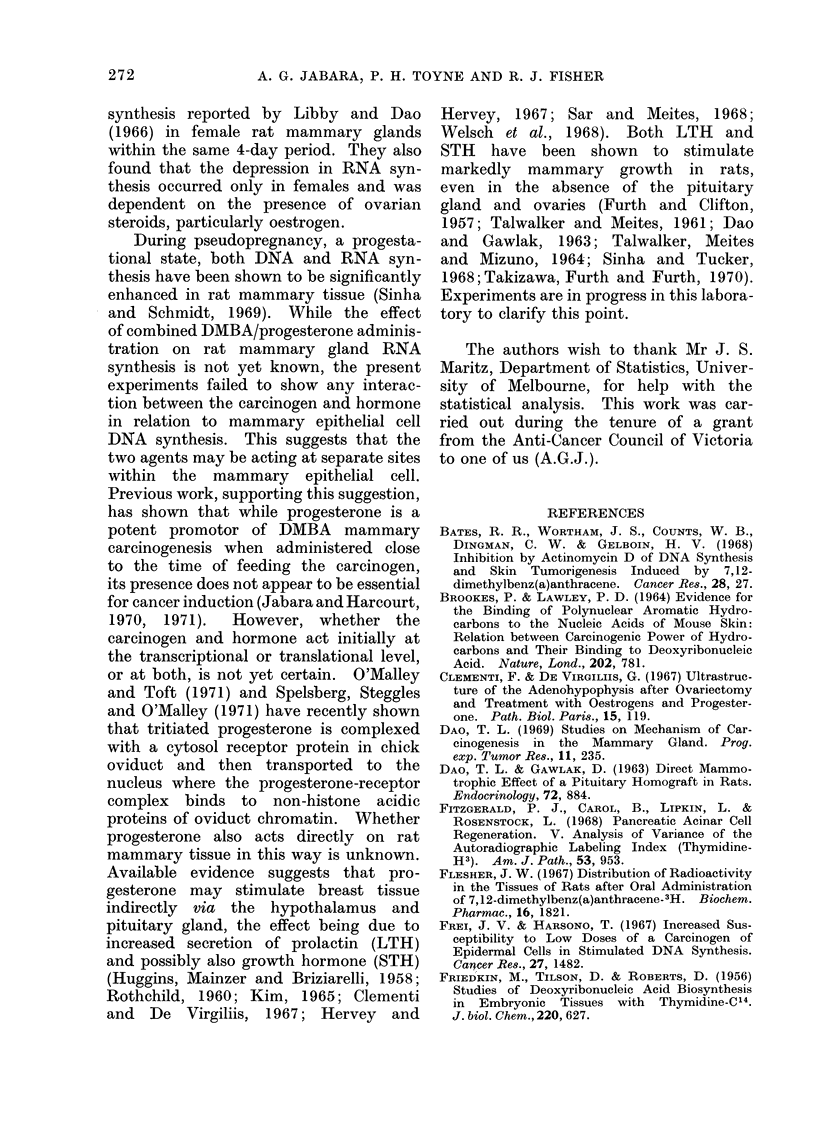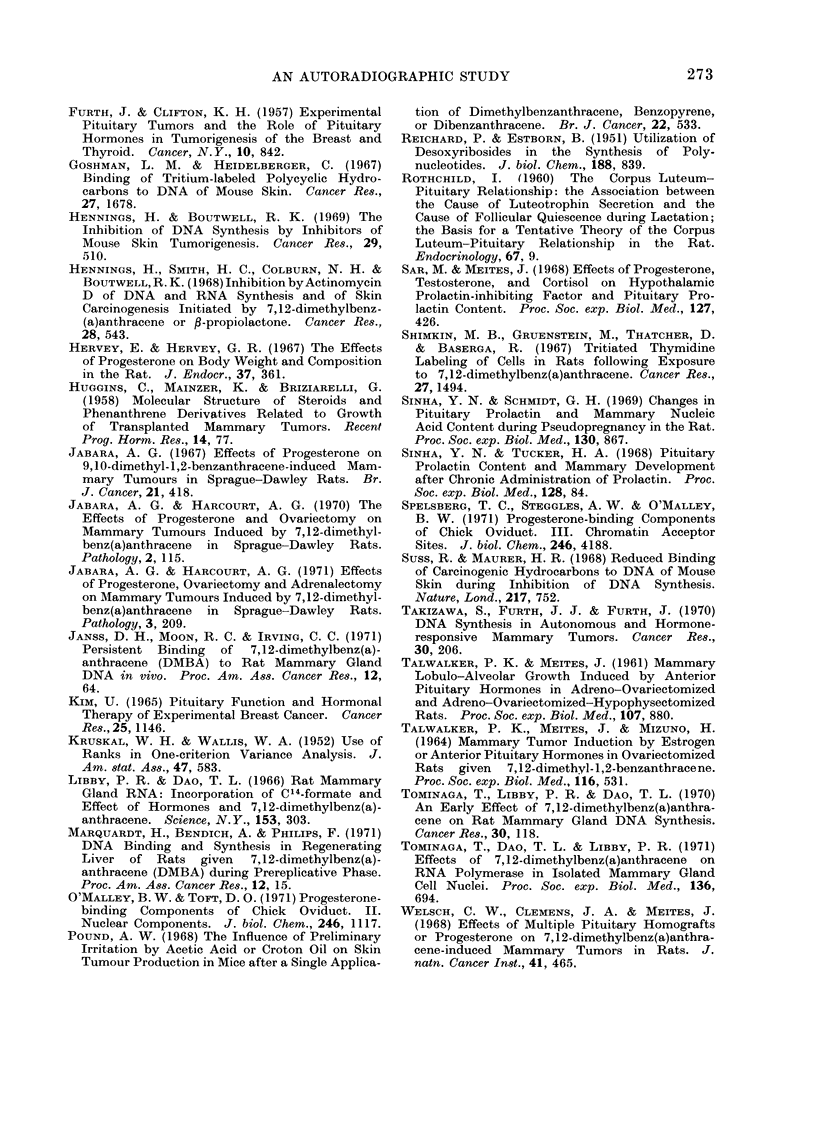# An Autoradiographic Study of the Early Effects of 7,12-Dimethylbenz(a)anthracene and Progesterone on DNA Synthesis in Rat Mammary Epithelial Cells and Subsequent Tumour Development

**DOI:** 10.1038/bjc.1972.36

**Published:** 1972-08

**Authors:** Anne G. Jabara, P. H. Toyne, R. J. Fischer

## Abstract

**Images:**


*Br. J. Cancer* (1972) **26**, 265.

### AN AUTORADIOGRAPHIC STUDY OF THE EARLY EFFECTS OF 7,12-DIMETHYLBENZ(a)ANTHRACENE AND PROGESTERONE ON DNA SYNTHESIS IN RAT MAMMARY EPITHELIAL CELLS AND SUBSEQUENT TUMOUR DEVELOPMENT

ANNE G. JABARA, P. H. TOYNE AND R. J. FISHER

*From the Departments of Pathology and Statistics, University of Melbourne, Parkville, 3052, Australia*

Received for publication March 1972

**Summary.—Experiments were undertaken to investigate the early effects of DMBA or progesterone, or the two in combination, on DNA synthesis in rat mammary epithelial cells, and also to determine whether there was any correlation between the level of DNA synthesis observed in the first 96 hours after administration of DMBA, either alone or combined with progesterone, and subsequent tumour development.**

**It was found that DMBA alone caused an insignificant reduction in DNA synthesis in the first 96 hours, whereas progesterone significantly enhanced DNA synthesis. When the carcinogen and hormone were administered together, a greater rise was seen in the level of DNA synthesis than that occurring in rats treated only with DMBA, but the increase was not significantly greater than that in untreated animals. A two-way analysis of variance revealed no interaction between DMBA and progesterone in relation to mammary epithelial cell DNA synthesis.**

**Mammary neoplasms occurred only in the groups of rats which had received DMBA, either alone or in combination with progesterone. No correlation could be demonstrated between the extent of DNA synthesis observed in mammary glands biopsied between 6 and 96 hours after carcinogen administration and the occurrence of tumours in the host rats 135 days later.**

PREVIOUS investigations have shown that exogenous progesterone, while not carcinogenic *per se*, significantly enhanced 7,12-dimethylbenz(a)anthracene (DMBA) mammary carcinogenesis in entire rats when hormone injections were begun either 2 days before or just after carcinogen administration (Jabara, 1967; Jabara and Harcourt, 1970). In contrast, Welsch, Clemens and Meites (1968) reported that prolonged (25 days) treatment with progesterone before administering DMBA significantly inhibited mammary tumorigenesis. They suggested that the enhanced mammary development induced by prolonged progesterone pretreatment rendered the gland relatively refractory to carcinogen action. DNA synthesis has been reported to be markedly depressed in rat mammary gland for at least 4 days after administration of DMBA alone (Shimkin *et al.*, 1967; Tominaga, Libby and Dao, 1970). Marquardt, Bendich and Philips (1971) have shown that while DMBA will bind to hepatocyte non-replicating DNA, the amount of bound DMBA increases three-fold during DNA synthesis, and Brookes and Lawley (1964) observed a close correlation between carcinogenicity and the amount of hydrocarbon bound to DNA in mouse skin. These observations suggest that the contrasting effects on DMBA mammary carcinogenesis of long or short pretreatment with progesterone might be related to the level of DNA synthesis in the glands at the time of feeding DMBA.

The present experiments were designed to determine (1) the early effects on DNA synthesis in mammary epithelial cells of DMBA, administered alone or combined with long or short pretreatment with progesterone, and (2) whether there was any correlation between the extent of DNA synthesis observed in the first 96 hours after administration of the carcinogen, either alone or combined with progesterone, and subsequent tumour development.

TABLE I.—*Average Number of Labelled Mammary Epithelial Nuclei per* 2000 *Cells per Control Rat* (*Group* 1) *in the Different Stages of the Oestrous Cycle*

| Oestrous cycle stage | Number of rats | Average number labelled nuclei per 2000 cells per rat ± S.D. |
|---|---|---|
| Pro-oestrus (stage 1) | 6 | 67±128·2 |
| Oestrus (stages 2 and 3) | 7 | 30±25·4 |
| Metoestrus (stage 4) | 5 | 50±51·9 |
| Dioestrus (stage 5) | 6 | 83±103·9 |

### MATERIALS AND METHODS

### *Treatment of animals*

One hundred and thirty-eight non-inbred Sprague–Dawley female rats aged 25 days (35–55 g body weight) were divided randomly into 6 groups (Tables I and II), housed 5 rats per cage and fed commercial pellets and water *ad libitum*. Rats in Group 1 served as untreated controls. Each animal in Groups 3–6 received daily subcutaneous injections of 3 mg of progesterone (Sigma Chemical Co., U.S.A.) dissolved in 0·1 ml of corn oil. In Groups 3 and 5, hormone injections were begun on their 25th day of age (P-25), and in Groups 4 and 6 on their 48th day of age (P-2), *i.e.* 25 and 2 days, respectively, before feeding the carcinogen; injections were continued until the predetermined times for removal of mammary tissue were reached (Table II). In addition, at 50 days of age each rat in Groups 5 and 6, as well as those in Group 2, was fed intragastrically a single 30 mg dose of DMBA (Eastman Organic Chemicals, U.S.A.) dissolved in 2 ml of corn oil (Table II). Daily vaginal smears were taken from all rats, beginning between their 45th and 50th day of age, for at least 2 complete oestrous cycles (Group 1) and for at least 3 complete cycles (Groups 2–6).

Two hours before removing the 2 most posterior mammary glands on the left side, 150 μCi of tritiated thymidine (Radiochemical Centre, Amersham) (specific activity 5·0 Ci/mmol/l) made up to 1 ml in sterile 0·9% saline solution was injected intraperitoneally. In Groups 2–6 the thymidine was injected one hour before the predetermined times of 6, 12, 24, 36, 48, 72 and 96 hours and the mammary tissue was removed one hour after these times. Mammary tissue was usually

TABLE II.—*Treatments Used and the Resulting Average Number of Labelled Mammary Epithelial Nuclei per* 2000 *Cells per Rat between* 6 *and* 96 *hours and the Group Average*

| | Group 1 | Group 2 | Group 3 | Group 4 | Group 5 | Group 6 |
|---|---|---|---|---|---|---|
| Group treatment | Nil | DMBA | P-25 | P-2 | DMBA + P-25 | DMBA + P-2 |
| Total no. rats | 24 | 25 | 21 | 21 | 23 | 24 |
| Removal of mammary tissue: | | | | | | |
| No. rats biopsied | 0 | 17 | 16 | 13 | 18 | 16 |
| No. rats autopsied | 24 | 8 | 5 | 8 | 5 | 8 |
| No. labelled nuclei per 2000 cells per rat* | | | | | | |
| 6 hours | — | 29 | 132 | 182 | 111 | 83 |
| 12 hours | — | 34 | 56 | 32 | 48 | 46 |
| 24 hours | — | 25 | 83 | 21 | 59 | 123 |
| 36 hours | — | 73 | 76 | 46 | 102 | 115 |
| 48 hours | — | 40 | 134 | 148 | 58 | 34 |
| 72 hours | — | 36 | 58 | 147 | 18 | 49 |
| 96 hours | — | 58† | 141 | 99 | 87‡ | 107§ |
| Group average | 57 | 44 | 97 | 96 | 70 | 83 |
| Within group standard deviation | 86·8 | 81·1 | 69·3 | 104·5 | 58·9 | 80·8 |

* Each time interval represents the average of 3 rats, except: † average of 7 rats, ‡ average of 5 rats, § average of 6 rats.

FIG. 1.—Autoradiograph of mammary tissue removed from a rat in Group 4 (P-2) at 48 hours (52 days of age), showing the presence of labelled nuclei in epithelial cells lining small ducts and alveoli. Kernechtrot/Tartrazine stain. × 600.

removed by surgical biopsy, but, as part of a preliminary experiment, some animals in each group were killed and the mammary tissue removed at autopsy (Table II). Biopsied rats were permitted to survive until their 185th day of age, *i.e.* 135 days after feeding the carcinogen. In the control animals, tritiated thymidine was injected intraperitoneally 2 hours before killing rats in the different stages of the oestrous cycle; the 2 most posterior mammary glands on the left side were removed at autopsy (Table I).

*Preparation of autoradiographs*

Excised mammary tissue was flattened on a thin card and fixed in 10% buffered formalin. Serial sections, 4 $\mu$m thick, were cut from each paraffin block and, to avoid counting the same cell twice, only every fourth section was used. Three sections per slide were mounted well towards one end of it, dried and deparaffinized. The slides were then dipped, under a Ilford safety light No. 904F, in Ilford K5 nuclear emulsion diluted 1 in 4 with metal distilled water and maintained at 46°C. After drying the slides for 1–2 hours in a black, dustproof, Perspex box, the slides were sealed in lightproof slide boxes (Clay–Adams Inc.) and exposed at 4°C. To allow for variation in labelling intensity, half the slides were developed in Agfa Neutol S after 20 days and the remainder after 40 days (Fig. 1). The sections were stained with 0·1% Kernechtrot (Chroma) in a 5% aluminium sulphate solution, counterstained with 2·5% tartrazine cellusolve (Chroma) and mounted in polystyrene.

*Examination of autoradiographs*

An adaptation of a random field method described by Fitzgerald *et al.* (1968) was used for counting the labelled and unlabelled mammary epithelial cells. The mammary tissue section was moved by the mechanical stage, while being viewed at low power, and the vernier numbers on the stage

FIG. 2.—A diagrammatic representation of a section of mammary tissue and overlying skin, showing the construction of a randomized set of lines which were then used for counting an autoradiograph. A new randomized set of lines was constructed for each tissue section counted.

corresponding to the lateral limits of the tissue were determined. The consecutive whole numbers falling between these 2 limits were then recorded (Fig. 2). A decimal point was added to the right of each whole number and a sequence of random numbers, drawn from a table of random numbers, was added successively to the right of each decimal point to form a randomized set of lines over the entire section (Fig. 2). Because the mammary tissue sections were long and narrow, unlike Fitzgerald *et al.* (1968) only one set of randomized lines was used, and all the epithelial cells encountered along each line were counted with the oil immersion objective. For each rat, 500 epithelial cells were counted from sections exposed for 20 days and, depending on the labelling intensity, a further 1500 cells from the same animal were counted from sections exposed for either 20 or 40 days; the number of labelled nuclei per 2000 cells per rat was recorded in 4 × 500 cells. An individual cell was considered to be labelled when at least 10 more silver grains were localized over the nucleus than were observed over an equivalent area of background; background labelling in almost all preparations was negligible.

*Treatment of tumour tissue*

Surviving animals in Groups 2–6 were sacrificed at 185 days of age. Portions of each tumour were removed at autopsy, fixed in 10% buffered formalin and 5 μm paraffin sections were stained with haematoxylin and eosin.

*Statistical methods*

For convenience, the sum of 4 estimations of the number of labelled epithelial nuclei per 500 cells is referred to as the LEN. The LEN for each rat is therefore an estimate of the number of labelled epithelial nuclei per 2000 cells.

An analysis of variance was used to test for the possible effects on the LEN of each of the following: stage of oestrous cycle, time of mammary biopsy, DMBA, progesterone, and DMBA combined with progesterone. For each of these analyses, the logarithm of the LEN for each rat was used because scatter diagrams suggested this to be the most appropriate transformation in stabilizing the variance of an observation. A variance analysis of ranked data (Kruskal and Wallis, 1952) was used to test for a possible relationship between the LEN and subsequent tumour occurrence and also to test for possible differences between treatment groups with respect to the average number of active tumour centres developed per rat. $\chi^2$ tests to contingency tables were used to analyse possible differences in tumour incidence and tumour multiplicity among different treatment groups.

### RESULTS

*Effect of oestrous cycle on LEN*

In the controls (Group 1) the average LEN per rat differed with the stage of the oestrous cycle, but these differences were not statistically significant, presumably due to the large variability of observations within each group (Table I). Therefore, in analyses of treated animals (Groups 2–6) the stage of the oestrous cycle was disregarded. At the time of mammary gland removal most of the treated rats were in dioestrus, except for 4 rats in Group 3 and one rat in Group 5 whose vaginae had not opened and 6 rats in Group 2 which were in either pro-oestrus or oestrus. Continuation of vaginal smearing for 2–3 weeks after taking mammary biopsies revealed that rats in Group 2 (DMBA only) continued to cycle normally in either a 4 or 5 day cycle, while animals in Groups 3–6, which had received progesterone up to the time of mammary gland removal, continued in dioestrus for approximately a further 10 days before they began to cycle normally.

*Effect of time of tissue sampling on LEN*

Within each group of treated rats (Groups 2–6) the LEN varied from one time interval to another (Table II), but these differences were not significant. The time of tissue sampling was therefore disregarded in other analyses.

*Effects of DMBA and progesterone on LEN*

The LEN (average of all rats in a group) for animals fed only DMBA (Group 2) was lower than that obtained for the controls (Group 1) and those obtained for rats receiving only progesterone (Groups 3 and 4) (Table II). Analysis revealed that while the difference between the LENs in the former 2 groups was not significant ($P > 0{\cdot}10$), the differences between Group 2 and each of the latter 2 progesterone-treated groups (3 and 4) were significant ($P < 0{\cdot}005$ and $P < 0{\cdot}025$, respectively). Progesterone significantly increased the LEN not only compared with that in the DMBA-treated rats, but also compared with the untreated controls (Group 1), regardless of whether injections were begun at 25 or 48 days of age ($P < 0{\cdot}01$ and $P < 0{\cdot}05$, respectively) (Table II). The difference between the LENs in the 2 hormone-treated groups (3 and 4) was not significant (Table II).

*Effects of combined DMBA/progesterone on LEN*

In the rats treated with both carcinogen and hormone, the LEN for Group 5 (DMBA + P-25) was lower, but not significantly so, than that for Group 6 (DMBA + P-2) (Table II), and neither of these LENs was significantly higher than that obtained for the controls (Group 1), the difference between the LENs for Group 1 and 6 just failing to reach significance at the 5% level ($P = 0{\cdot}051$). However, the LENs for Groups 5 and 6 were both significantly higher than that obtained for Group 2 (DMBA only) ($P < 0{\cdot}01$ and $P < 0{\cdot}025$, respectively) (Table II). The LEN for Group 5 (DMBA + P-25) was not significantly different from that obtained in Group 3 (P-25), and, similarly, the LEN for Group 6 (DMBA + P-2) was not significantly different from that obtained in Group 4 (P-2) (Table II). The possibility of an interaction between DMBA and progesterone in relation to the LEN was tested by means of a two-way analysis of variance. The result was not significant ($P > 0{\cdot}10$), indicating that the effect due to DMBA did not appear to vary with the presence or absence of progesterone, and likewise the relative effect due to progesterone appeared to be independent of the presence or absence of DMBA.

*Effects of treatment regimens on tumour yield*

Mammary tumours arose only in Groups 2, 5 and 6 which had received DMBA, either alone or in combination

TABLE III.—*Treatments Used and the Resulting Mammary Tumour Incidences, Number of Rats with Multiple Tumours and Histological Tumour Types Induced*

| Group treatment | Group 1 Nil | Group 2 DMBA | Group 3 P-25 | Group 4 P-2 | Group 5 DMBA + P-25 | Group 6 DMBA + P-2 |
|---|---|---|---|---|---|---|
| Survivors after mammary gland removal | 0 | 17 | 16 | 13 | 18 | 16 |
| Survivors at end of experiment | 0 | 12 | 16 | 13 | 17 | 16 |
| No. of rats with tumours | — | 6 | 0 | 0 | 7 | 9 |
| (percentage) | — | (50·0) | — | — | (41·2) | (56·3) |
| Total no. of tumours | — | 17 | 0 | 0 | 10 | 24 |
| No. of rats with multiple* tumours | — | 4 | 0 | 0 | 2 | 6 |
| (percentage) | — | (33·3) | — | — | (11·8) | (37·5) |
| Average no. of active tumour centres per rat | — | 1·4 | 0 | 0 | 0·6 | 1·5 |
| Histological tumour types | | | | | | |
| No. classified carcinoma | — | 17 | 0 | 0 | 10 | 23 |
| No. classified adenoma | — | 0 | 0 | 0 | 0 | 1 |

* Two or more tumours per rat.

with progesterone (Table III). All biopsied rats within these 3 groups survived, with the exception of 5 animals in Group 2 which died 55 days after carcinogen administration, due to a failure in the automatic watering system over a holiday period, and one rat in Group 5 which died from pneumonitis 105 days after receiving DMBA. None of these 6 rats bore palpable tumours at death and, from a previous series, these times were considered to be too short for the development of palpable neoplasms (unpublished data). Hence, all figures and statistics relating to tumour yield have been based only on the rats which survived to the end of the experiment (135 days after feeding DMBA).

The tumour incidence, numbers of rats developing multiple tumours and the average number of active tumour centres developed per rat varied between the 3 groups, appearing lowest in Group 5 (Table III), but none of these differences was statistically significant.

*Relationship between LEN and tumour development*

To test for a possible relationship between the LEN and subsequent tumour occurrence, the rats were divided into 2 groups (a) those with a LEN of less than 40 and (b) those with a LEN of greater than 40, and in addition the rats were paired into identical time and treatment groups in an attempt to eliminate any effect due to these two variables. This necessitated randomly deleting 17 of the 45 rats in Groups 2, 5 and 6 from the analysis. The Kruskal and Wallis (1952) test demonstrated no evidence of any relationship between the LEN observed in mammary glands biopsied between 6 and 96 hours after DMBA administration and the observed occurrence of tumours in the host rats (Groups 2, 5 and 6) 135 days later ($P > 0{\cdot}10$).

With the exception of an adenoma arising in one rat in Group 6, histologically all the neoplasms were carcinomata (Table III), the majority being adenocarcinomata and the remainder of a solid, poorly differentiated type (Jabara, 1967).

### DISCUSSION

Thymidine is incorporated only into cells undergoing DNA synthesis (Reichard and Estborn, 1951; Friedkin, Tilson and Roberts, 1956), so that the number of cells labelled with tritiated thymidine per 2000 gives an estimate of the number of cells undergoing DNA synthesis at the particular time of tissue sampling. Shimkin *et al.* (1967) and Tominaga, Libby and Dao (1970) reported a significant depression in DNA synthesis in mammary epithelial cells up to 96 hours after feeding DMBA. In the present series, there was a trend towards a depression in mammary epithelial DNA synthesis between 6 and 96 hours following administration of DMBA alone, but the level of DNA synthesis was not significantly reduced below control levels. Analysis of the 3 experiments revealed no obvious reason for this difference, and the explanation may possibly lie in the use of a more resistant substrain of Sprague–Dawley rat in the present series.

In contrast to DMBA, progesterone significantly increased DNA synthesis in the 6–96 hour period above control levels, regardless of whether injections were begun at 25 or 48 days of age. Similarly, administration of DMBA combined with either long or short progesterone pretreatment, resulted in an increase in DNA synthesis above both control (an insignificant rise) and DMBA values (a significant increase). Furthermore, a subjective histological assessment of mammary development in the 6 groups revealed that although there was considerable individual variation in the extent of lobular–alveolar development, glandular development was similar in the 4 progesterone-treated groups, and was greater than that seen in the glands from the controls and DMBA-treated rats. The similarity in both the level of DNA synthesis and the extent of mammary gland development in rats given long or short progesterone treatments before feeding DMBA, fails to confirm the suggestion advanced by Welsch, Clemens and Meites (1968) that enhanced mammary development renders the gland refractory to DMBA and results in decreased tumorigenesis following prolonged progesterone pretreatment. In the present series, there is a trend towards a lower overall tumour yield in Group 5 (DMBA + P-2), but the difference is not significant. Dao (1969) has suggested that under strong hormonal stimulation interaction between polycyclic hydrocarbons and hyperfunctioning mammary epithelial cells may be inhibited. It would be of interest to investigate the amounts of DMBA bound to mammary epithelial cell macromolecules derived from virgin, pregnant and progesterone-treated animals.

Several studies on mouse skin have suggested that DNA synthesis may be important in DMBA carcinogenesis (Frei and Harsono, 1967; Bates *et al.*, 1968; Hennings *et al.*, 1968; Pound, 1968; Suss and Maurer, 1968). Other investigations, however, have been unable to demonstrate a direct relationship between the early inhibitory effect of DMBA on DNA synthesis and tumour yield in mouse skin (Goshman and Heidelberger, 1967; Hennings and Boutwell, 1969), and a similar conclusion has been reached in relation to DMBA mammary carcinogenesis (Shimkin *et al.*, 1967; Tominaga *et al.*, 1970). In the latter 2 experiments, the conclusion was based on the finding that DMBA decreased tritiated thymidine incorporation into mammary gland DNA not only in females but also in male rats, and yet, unlike females, male rats rarely develop mammary neoplasms following administration of a single dose of the carcinogen. Findings in the present series not only confirm this conclusion, but further suggest that no direct relationship exists between DNA synthesis and subsequent tumour yield, regardless of whether DNA synthesis is increased or decreased close to the time of DMBA administration.

In rat mammary gland and mouse skin, DMBA binds not only to DNA but also to RNA and proteins (Flesher, 1967; Goshman and Heidelberger, 1967; Janss, Moon and Irving, 1971), and recent work suggests that early events in mammary carcinogenesis may be related to the effects of DMBA on DNA-dependent RNA polymerase activity (Tominaga *et al.*, 1971). These investigators observed a significant decrease in activity of this enzyme for 2 days after DMBA administration to female rats, and activity then increased to a level significantly above control values by 4 days. No depression in RNA polymerase activity occurred in male rats fed DMBA. These findings substantiate the alterations in RNA

synthesis reported by Libby and Dao (1966) in female rat mammary glands within the same 4-day period. They also found that the depression in RNA synthesis occurred only in females and was dependent on the presence of ovarian steroids, particularly oestrogen.

During pseudopregnancy, a progestational state, both DNA and RNA synthesis have been shown to be significantly enhanced in rat mammary tissue (Sinha and Schmidt, 1969). While the effect of combined DMBA/progesterone administration on rat mammary gland RNA synthesis is not yet known, the present experiments failed to show any interaction between the carcinogen and hormone in relation to mammary epithelial cell DNA synthesis. This suggests that the two agents may be acting at separate sites within the mammary epithelial cell. Previous work, supporting this suggestion, has shown that while progesterone is a potent promotor of DMBA mammary carcinogenesis when administered close to the time of feeding the carcinogen, its presence does not appear to be essential for cancer induction (Jabara and Harcourt, 1970, 1971). However, whether the carcinogen and hormone act initially at the transcriptional or translational level, or at both, is not yet certain. O'Malley and Toft (1971) and Spelsberg, Steggles and O'Malley (1971) have recently shown that tritiated progesterone is complexed with a cytosol receptor protein in chick oviduct and then transported to the nucleus where the progesterone-receptor complex binds to non-histone acidic proteins of oviduct chromatin. Whether progesterone also acts directly on rat mammary tissue in this way is unknown. Available evidence suggests that progesterone may stimulate breast tissue indirectly *via* the hypothalamus and pituitary gland, the effect being due to increased secretion of prolactin (LTH) and possibly also growth hormone (STH) (Huggins, Mainzer and Briziarelli, 1958; Rothchild, 1960; Kim, 1965; Clementi and De Virgiliis, 1967; Hervey and Hervey, 1967; Sar and Meites, 1968; Welsch *et al.*, 1968). Both LTH and STH have been shown to stimulate markedly mammary growth in rats, even in the absence of the pituitary gland and ovaries (Furth and Clifton, 1957; Talwalker and Meites, 1961; Dao and Gawlak, 1963; Talwalker, Meites and Mizuno, 1964; Sinha and Tucker, 1968; Takizawa, Furth and Furth, 1970). Experiments are in progress in this laboratory to clarify this point.

The authors wish to thank Mr J. S. Maritz, Department of Statistics, University of Melbourne, for help with the statistical analysis. This work was carried out during the tenure of a grant from the Anti-Cancer Council of Victoria to one of us (A.G.J.).

### REFERENCES

Bates, R. R., Wortham, J. S., Counts, W. B., Dingman, C. W. & Gelboin, H. V. (1968) Inhibition by Actinomycin D of DNA Synthesis and Skin Tumorigenesis Induced by 7,12-dimethylbenz(a)anthracene. *Cancer Res.*, **28**, 27.

Brookes, P. & Lawley, P. D. (1964) Evidence for the Binding of Polynuclear Aromatic Hydrocarbons to the Nucleic Acids of Mouse Skin: Relation between Carcinogenic Power of Hydrocarbons and Their Binding to Deoxyribonucleic Acid. *Nature, Lond.*, **202**, 781.

Clementi, F. & De Virgiliis, G. (1967) Ultrastructure of the Adenohypophysis after Ovariectomy and Treatment with Oestrogens and Progesterone. *Path. Biol. Paris.*, **15**, 119.

Dao, T. L. (1969) Studies on Mechanism of Carcinogenesis in the Mammary Gland. *Prog. exp. Tumor Res.*, **11**, 235.

Dao, T. L. & Gawlak, D. (1963) Direct Mammotrophic Effect of a Pituitary Homograft in Rats. *Endocrinology*, **72**, 884.

Fitzgerald, P. J., Carol, B., Lipkin, L. & Rosenstock, L. (1968) Pancreatic Acinar Cell Regeneration. V. Analysis of Variance of the Autoradiographic Labeling Index (Thymidine-$H^3$). *Am. J. Path.*, **53**, 953.

Flesher, J. W. (1967) Distribution of Radioactivity in the Tissues of Rats after Oral Administration of 7,12-dimethylbenz(a)anthracene-$^3H$. *Biochem. Pharmac.*, **16**, 1821.

Frei, J. V. & Harsono, T. (1967) Increased Susceptibility to Low Doses of a Carcinogen of Epidermal Cells in Stimulated DNA Synthesis. *Cancer Res.*, **27**, 1482.

Friedkin, M., Tilson, D. & Roberts, D. (1956) Studies of Deoxyribonucleic Acid Biosynthesis in Embryonic Tissues with Thymidine-$C^{14}$. *J. biol. Chem.*, **220**, 627.

FURTH, J. & CLIFTON, K. H. (1957) Experimental Pituitary Tumors and the Role of Pituitary Hormones in Tumorigenesis of the Breast and Thyroid. *Cancer, N.Y.*, **10**, 842.

GOSHMAN, L. M. & HEIDELBERGER, C. (1967) Binding of Tritium-labeled Polycyclic Hydrocarbons to DNA of Mouse Skin. *Cancer Res.*, **27**, 1678.

HENNINGS, H. & BOUTWELL, R. K. (1969) The Inhibition of DNA Synthesis by Inhibitors of Mouse Skin Tumorigenesis. *Cancer Res.*, **29**, 510.

HENNINGS, H., SMITH, H. C., COLBURN, N. H. & BOUTWELL, R. K. (1968) Inhibition by Actinomycin D of DNA and RNA Synthesis and of Skin Carcinogenesis Initiated by 7,12-dimethylbenz-(a)anthracene or β-propiolactone. *Cancer Res.*, **28**, 543.

HERVEY, E. & HERVEY, G. R. (1967) The Effects of Progesterone on Body Weight and Composition in the Rat. *J. Endocr.*, **37**, 361.

HUGGINS, C., MAINZER, K. & BRIZIARELLI, G. (1958) Molecular Structure of Steroids and Phenanthrene Derivatives Related to Growth of Transplanted Mammary Tumors. *Recent Prog. Horm. Res.*, **14**, 77.

JABARA, A. G. (1967) Effects of Progesterone on 9,10-dimethyl-1,2-benzanthracene-induced Mammary Tumours in Sprague–Dawley Rats. *Br. J. Cancer*, **21**, 418.

JABARA, A. G. & HARCOURT, A. G. (1970) The Effects of Progesterone and Ovariectomy on Mammary Tumours Induced by 7,12-dimethylbenz(a)anthracene in Sprague–Dawley Rats. *Pathology*, **2**, 115.

JABARA, A. G. & HARCOURT, A. G. (1971) Effects of Progesterone, Ovariectomy and Adrenalectomy on Mammary Tumours Induced by 7,12-dimethylbenz(a)anthracene in Sprague–Dawley Rats. *Pathology*, **3**, 209.

JANSS, D. H., MOON, R. C. & IRVING, C. C. (1971) Persistent Binding of 7,12-dimethylbenz(a)-anthracene (DMBA) to Rat Mammary Gland DNA *in vivo*. *Proc. Am. Ass. Cancer Res.*, **12**, 64.

KIM, U. (1965) Pituitary Function and Hormonal Therapy of Experimental Breast Cancer. *Cancer Res.*, **25**, 1146.

KRUSKAL, W. H. & WALLIS, W. A. (1952) Use of Ranks in One-criterion Variance Analysis. *J. Am. stat. Ass.*, **47**, 583.

LIBBY, P. R. & DAO, T. L. (1966) Rat Mammary Gland RNA: Incorporation of $C^{14}$-formate and Effect of Hormones and 7,12-dimethylbenz(a)-anthracene. *Science, N.Y.*, **153**, 303.

MARQUARDT, H., BENDICH, A. & PHILIPS, F. (1971) DNA Binding and Synthesis in Regenerating Liver of Rats given 7,12-dimethylbenz(a)-anthracene (DMBA) during Prereplicative Phase. *Proc. Am. Ass. Cancer Res.*, **12**, 15.

O'MALLEY, B. W. & TOFT, D. O. (1971) Progesterone-binding Components of Chick Oviduct. II. Nuclear Components. *J. biol. Chem.*, **246**, 1117.

POUND, A. W. (1968) The Influence of Preliminary Irritation by Acetic Acid or Croton Oil on Skin Tumour Production in Mice after a Single Application of Dimethylbenzanthracene, Benzopyrene, or Dibenzanthracene. *Br. J. Cancer*, **22**, 533.

REICHARD, P. & ESTBORN, B. (1951) Utilization of Desoxyribosides in the Synthesis of Polynucleotides. *J. biol. Chem.*, **188**, 839.

ROTHCHILD, I. (1960) The Corpus Luteum–Pituitary Relationship: the Association between the Cause of Luteotrophin Secretion and the Cause of Follicular Quiescence during Lactation; the Basis for a Tentative Theory of the Corpus Luteum–Pituitary Relationship in the Rat. *Endocrinology*, **67**, 9.

SAR, M. & MEITES, J. (1968) Effects of Progesterone, Testosterone, and Cortisol on Hypothalamic Prolactin-inhibiting Factor and Pituitary Prolactin Content. *Proc. Soc. exp. Biol. Med.*, **127**, 426.

SHIMKIN, M. B., GRUENSTEIN, M., THATCHER, D. & BASERGA, R. (1967) Tritiated Thymidine Labeling of Cells in Rats following Exposure to 7,12-dimethylbenz(a)anthracene. *Cancer Res.*, **27**, 1494.

SINHA, Y. N. & SCHMIDT, G. H. (1969) Changes in Pituitary Prolactin and Mammary Nucleic Acid Content during Pseudopregnancy in the Rat. *Proc. Soc. exp. Biol. Med.*, **130**, 867.

SINHA, Y. N. & TUCKER, H. A. (1968) Pituitary Prolactin Content and Mammary Development after Chronic Administration of Prolactin. *Proc. Soc. exp. Biol. Med.*, **128**, 84.

SPELSBERG, T. C., STEGGLES, A. W. & O'MALLEY, B. W. (1971) Progesterone-binding Components of Chick Oviduct. III. Chromatin Acceptor Sites. *J. biol. Chem.*, **246**, 4188.

SUSS, R. & MAURER, H. R. (1968) Reduced Binding of Carcinogenic Hydrocarbons to DNA of Mouse Skin during Inhibition of DNA Synthesis. *Nature, Lond.*, **217**, 752.

TAKIZAWA, S., FURTH, J. J. & FURTH, J. (1970) DNA Synthesis in Autonomous and Hormone-responsive Mammary Tumors. *Cancer Res.*, **30**, 206.

TALWALKER, P. K. & MEITES, J. (1961) Mammary Lobulo–Alveolar Growth Induced by Anterior Pituitary Hormones in Adreno–Ovariectomized and Adreno–Ovariectomized–Hypophysectomized Rats. *Proc. Soc. exp. Biol. Med.*, **107**, 880.

TALWALKER, P. K., MEITES, J. & MIZUNO, H. (1964) Mammary Tumor Induction by Estrogen or Anterior Pituitary Hormones in Ovariectomized Rats given 7,12-dimethyl-1,2-benzanthracene. *Proc. Soc. exp. Biol. Med.*, **116**, 531.

TOMINAGA, T., LIBBY, P. R. & DAO, T. L. (1970) An Early Effect of 7,12-dimethylbenz(a)anthracene on Rat Mammary Gland DNA Synthesis. *Cancer Res.*, **30**, 118.

TOMINAGA, T., DAO, T. L. & LIBBY, P. R. (1971) Effects of 7,12-dimethylbenz(a)anthracene on RNA Polymerase in Isolated Mammary Gland Cell Nuclei. *Proc. Soc. exp. Biol. Med.*, **136**, 694.

WELSCH, C. W., CLEMENS, J. A. & MEITES, J. (1968) Effects of Multiple Pituitary Homografts or Progesterone on 7,12-dimethylbenz(a)anthracene-induced Mammary Tumors in Rats. *J. natn. Cancer Inst.*, **41**, 465.